# Enhanced differentiation of human pluripotent stem cells into pancreatic progenitors co-expressing PDX1 and NKX6.1

**DOI:** 10.1186/s13287-017-0759-z

**Published:** 2018-01-23

**Authors:** Bushra Memon, Manale Karam, Sara Al-Khawaga, Essam M. Abdelalim

**Affiliations:** 1 Diabetes Research Center, Qatar Biomedical Research Institute, Hamad Bin Khalifa University, Qatar Foundation, Doha, Qatar; 2 Cancer Research Center, Qatar Biomedical Research Institute, Hamad Bin Khalifa University, Qatar Foundation, Doha, Qatar

**Keywords:** hPSCs, Beta cells, Diabetes, Differentiation, Transcription factors, Pancreatic epithelium

## Abstract

**Background:**

Pancreatic progenitors (PPs) co-expressing the two transcription factors (TFs) PDX1 and NKX6.1 are recognized as the indispensable precursors of functional pancreatic β cells. Here, we aimed to establish an efficient protocol for maximizing generation of PDX1^+^/NKX6.1^+^ PPs from human pluripotent stem cells (hPSCs).

**Methods:**

In order to enhance the PDX1^+^/NKX6.1^+^ population, we manipulated in vitro culture conditions during differentiation by dissociating densely formed endodermal cells and re-plating them at different densities. These dissociated cells were subjected to an augmented duration of retinoid and fibroblast growth factor (FGF)10 signaling to induce higher PDX1 and NKX6.1 expression.

**Results:**

Our optimized protocol dramatically increased the expression of NKX6.1, leading to an increase in the proportion of PDX1^+^/NKX6.1^+^ progenitors (~90%) in monolayer, higher than the previously published protocols, as well as upregulated key TFs controlling pancreatic development. The improved efficiency of pancreatic differentiation was complemented by an inhibited hepatic specification and an increased proliferation of NKX6.1^+^ cells. Interestingly, we were able to enrich a novel PDX1^–^/NKX6.1^+^ population by manipulating the re-plating density; these oriented themselves in three-dimensional clusters. Further differentiation validated the ability of our PDX1^+^/NKX6.1^+^ progenitors to generate NGN3^+^ endocrine progenitors.

**Conclusions:**

We provide a novel technique that facilitates appropriate cellular rearrangement in monolayer culture to yield a high proportion of PDX1^+^/NKX6.1^+^ PPs with an elevated self-replicating capacity, thereby aiding scalable production of functional β cells from hPSCs in vitro. Our innovative method also enriches a novel NKX6.1^+^/PDX1^–^ population, with characteristics of proposed endocrine precursors, allowing further studies on deciphering routes to β-cell development.

**Electronic supplementary material:**

The online version of this article (doi:10.1186/s13287-017-0759-z) contains supplementary material, which is available to authorized users.

## Background

Diabetes is a globally widespread disease that exists in two major forms: type 1 diabetes (T1D) and type 2 diabetes (T2D). Both forms of this disease are characterized by loss of pancreatic β cells. T1D is characterized by autoimmune destruction of insulin-producing β cells of the pancreas, whereas in T2D pancreatic β-cell failure is a result of β-cell exhaustion after hypersecretion of insulin to overcome insulin resistance [1]. To date, the pathogenesis of diabetes is poorly understood and, as a consequence, there is no current permanent cure for this disease. Therefore, alternatively, researchers are actively exploring strategies to generate functional pancreatic β cells for potential cell replacement therapy as well as for disease modeling of diabetes. Human pluripotent stem cells (hPSCs) can recapitulate human pancreatic development to generate pancreatic progenitors that can be further differentiated into insulin-secreting β cells. Therefore, hPSC-derived pancreatic cells have a great potential to be used for diabetes treatment [2]. Step-wise protocols have been designed to differentiate hPSCs into β cells by directing them along the stages of definitive endoderm, pancreatic foregut, pancreatic progenitors, and endocrine precursor cells that finally mature into insulin-secreting cells [3–9]. These protocols involve the use of specific growth factors or pharmacological molecules that regulate specific signaling pathways. This is marked by the reconstruction of crucial human developmental cues that include activation or inhibition of appropriate transcription factors (TFs) and alternative signaling pathways [3–9].

Notably, differentiating hPSCs into pancreatic progenitors that co-express a panel of markers indispensable for inducing a β-cell fate is a key, decisive step for in vitro generation of β cells. Differentiation of the definitive endoderm (DE) into pancreatic progenitors is controlled by pancreatic and duodenal homeobox 1 (PDX1) TF which promotes pancreatic differentiation in concert with other TFs, such as NK6 homeobox transcription factor-related locus 1 (NKX6.1) [10]. When allowed to mature in vivo, NKX6.1-enriched pancreatic progenitors generated a higher proportion of functional insulin-secreting β cells compared with progenitors that had low expression of NKX6.1 [7–9, 11], indicating that the expression of NKX6.1 in pancreatic progenitors determines the functionality of β cells [12]. On the other hand, PDX1^+^/NKX6.1^–^ cells differentiate into poly-hormonal or glucagon-secreting cells [13]. Therefore, a high co-expression of PDX1 and NKX6.1 in pancreatic progenitors is crucial for an efficient induction of the endocrine progenitors, marked by the expression of Neurogenin 3 (NGN3), that will specifically generate functional insulin-secreting β cells. Efforts towards inducing the above regulatory TFs at appropriate stages of directed differentiation of hPSCs have allowed a few groups to successfully generate functional, mono-hormonal insulin-secreting β cells in vitro [7–9].

While the functionality and efficiency of in vitro generated β cells is widely debated [7–10, 14–16], pancreatic progenitors co-expressing PDX1 and NKX6.1 are currently employed in clinical trial for evaluating the safety and efficacy of their therapeutic use in treating T1D [17] (http://viacyte.com/clinical/clinical-trials/). Nevertheless, to encounter the issue of scaling up the production of hPSC-derived pancreatic β cells, optimization of in vitro protocols that generate a high yield of the PDX1^+^/NKX6.1^+^ population and enhance their proliferative capacity is needed to accelerate their clinical use. While much focus has been assigned to determining the appropriate cytokine cocktail to mimic in vivo development [5, 18, 19], the impact of modulating in vitro culture conditions that affect the cell’s physical environment, such as plating density, cell-cell contact, and properties of the extracellular matrix (ECM), on pancreatic differentiation is less well studied [20–26].

More recently, Nostro et al. provided the optimum temporal window and cytokine cocktail for higher induction of NKX6.1 in pancreatic progenitors in adherent culture [19]. Another group showed the upregulating effect of high-density aggregate cultures on PDX1^+^/NKX6.1^+^ pancreatic progenitors [27, 28]. Herein, we present an efficient method for producing a high proportion of PDX1^+^/NKX6.1^+^ pancreatic progenitors from hPSCs and maximizing their proliferative capacity. We show that dissociation and re-plating of endodermal cells at half their density in monolayer culture, followed by an extended duration of retinoid and fibroblast growth factor (FGF) signaling, specifically promotes NKX6.1 expression. Interestingly, our method also enriched a novel NKX6.1^+^ population, devoid of PDX1 expression, that may be a new source of pancreatic β cells.

## Methods

### Culture of human pluripotent stem cells

The H1 human embryonic stem cells (H1-hESCs) and IMR90-hiPSCs were obtained from WiCell Research Institute (Madison, WI, USA). Both cell lines were maintained in mTesR1 medium (Stem Cell Technologies, Canada) on Matrigel-coated dishes (Corning, USA; Matrigel was diluted at a concentration of 1:80 in knockout DMEM). Cells were passaged when they reached above 70% confluency by detachment with ReLeSR (Stem Cell Technologies, Canada) and resuspended in mTesR1 containing 10 μM Y-27632 (Rock inhibitor; Stemgent, USA). Cells were supplemented with fresh mTesR1 every day.

### Differentiation of human pluripotent stem cells into pancreatic progenitors

**Fig. 1:**
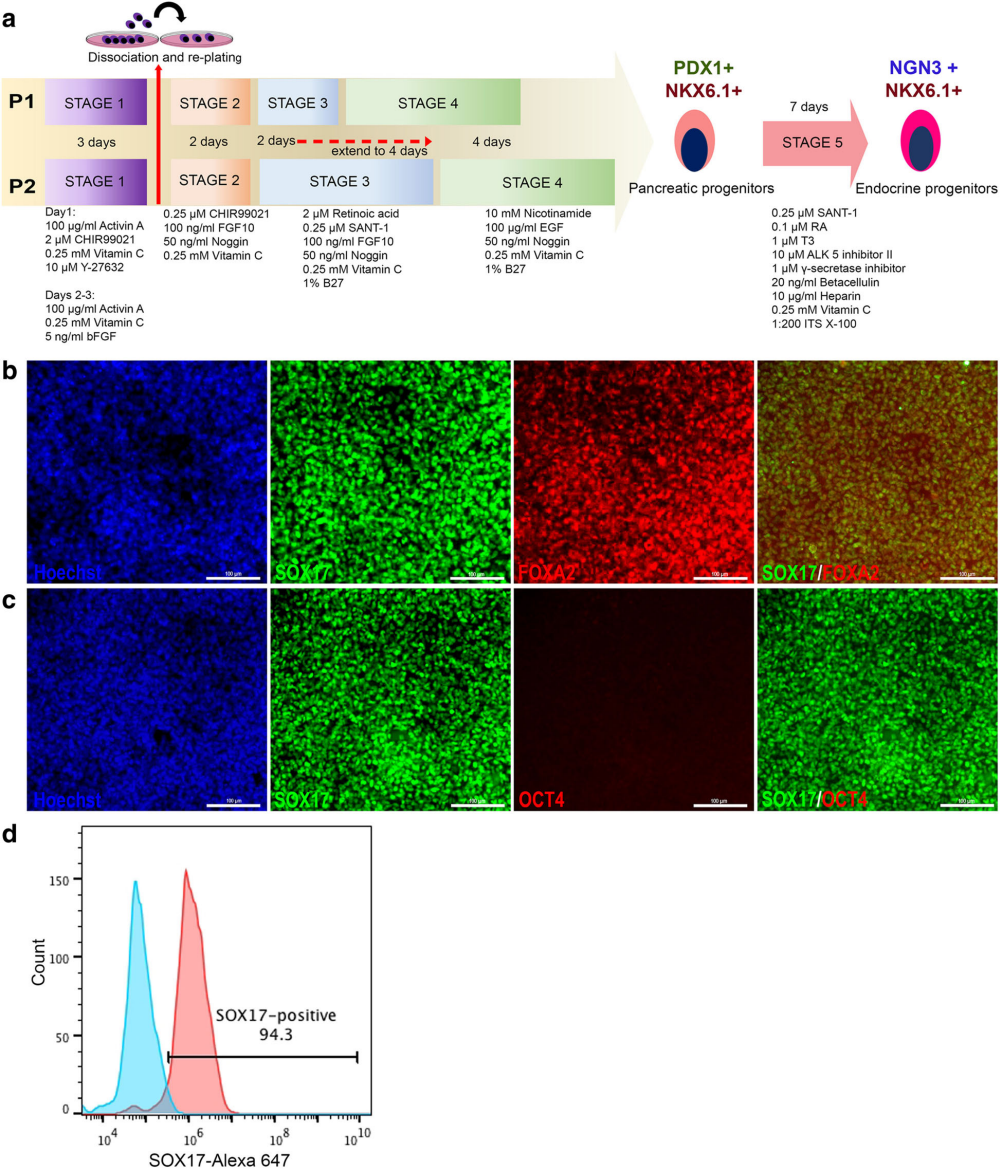
**a** Schematic overview of the protocols used for the differentiation of hPSCs into pancreatic progenitors. hPSCs are differentiated through the stages of definitive endoderm (DE) and posterior foregut to yield pancreatic progenitors through two different protocols differing in their duration of stage 3 treatment. Each of these protocols were either dissociated after generation of DE or left unmanipulated throughout the differentiation stages. Pancreatic progenitors generated using protocol 2 (P2) following dissociation were further differentiated through stage 5 to generate endocrine progenitors in adherent culture. **b** Representative immunofluorescent images of hESC-derived DE expressing high levels of SOX17 (green) and FOXA2 (red) after stage 1 of differentiation. The differentiated cells lost their pluripotency as indicated by the loss of OCT4 at the end of stage 1 (**c**). Nuclei are labeled with Hoechst. **d** Flow cytometry analysis of SOX17 expression at the end of stage 1 of differentiation. All data shown are representative results from at least three independent experiments. Scale bars = 100 μm

hPSCs were differentiated to pancreatic progenitors (PPs) using two previously published protocols with slight modifications in media composition [19]. hPSCs differentiated using the two protocols were either dissociated and re-plated following formation of endoderm or left unmanipulated (Fig. 1a). Differentiation was initiated when hPSCs reached 60–75% confluency. mTesR1 media was replaced with MCDB 131 (ThermoFisher Scientific, USA) composed of 2 mM glutamax, 0.5% fatty acid free bovine serum albumin (BSA; Sigma, USA), 1.5 g/L NaHCO_3_ (VWR, USA) and 1% penicillin/streptomycin as basal media and supplemented with 100 ng/ml hActivin A (R&D Systems, MN, USA), 2 μM CHIR99021 (Stemgent, USA), 0.25 mM vitamin C (Sigma, USA), and 10 μM Y-27632 (Rock inhibitor; Stemgent, USA) for day 1 of differentiation (stage 1 day 1). For days 2–3, the basal media were supplemented with 100 ng/ml hActivin A, 0.25 mM vitamin C, and 5 ng/ml basic fibroblast growth factor (bFGF; Stem Cell Technologies, USA). On day 4 (stage 2 day 1), the cells for each protocol were either dissociated using TrypLE and re-plated on fresh Matrigel (1:50) coated dishes or left adherent. Basal media as prepared in stage 1 were supplemented with 0.25 mM vitamin C, 50 ng/ml hFGF10 (R&D Systems, USA), 0.25 μM CHIR99021, and 50 ng/ml NOGGIN (R&D Systems, USA) for 2 days of stage 2 (days 4–5). For all experiments, except when stated otherwise, the dissociated cells were re-plated at densities within the range 2.5–3.5 × 10^5^ cells/cm^2^, which was about half the endoderm density for the individual experiments. For Protocol 1 (P1; both dissociated (D) and non-dissociated (ND)), stage 3 treatment was performed for 2 days, while for Protocol 2 (P2; both dissociated (D) and non-dissociated (ND)) this was performed for 4 days. For stage 3 differentiation, DMEM (ThermoFisher Scientific, USA) was supplemented with 1% penicillin/streptomycin, 1% vol/vol B27 supplement without vitamin A (ThermoFisher Scientific, USA), 0.25 mM vitamin C, 50 ng/ml hFGF10, 50 ng/ml hNOGGIN, 2 μM retinoic acid (Sigma, USA), and 0.25 μM SANT-1 (Sigma, USA) for the specified number of days for each protocol. At the end of stage 3, media were changed to DMEM supplemented with 1% vol/vol B27, 0.25 mM vitamin C, 50 ng/ml hFGF10, 50 ng/ml hNOGGIN, 100 ng/ml hEGF, and 10 mM nicotinamide (Sigma, USA) for 4 days of stage 4 treatment for each protocol (Fig. 1a). For generation of endocrine progenitors (stage 5), P2-D pancreatic progenitors were washed twice with DPBS at the end of stage 4 and supplemented with stage 5 day 1 media as specified by Pagliuca et al. [8] under adherent condition. Stage 5 treatment was performed for 7 days.

### Immunostaining

Differentiated hPSCs derived using different protocols were washed twice with phosphate-buffered saline (PBS) and fixed in 4% paraformaldehyde in 0.1 M phosphate buffer (pH 7.4) for 20 min. The cells were permeabilized for 15 min with 0.4% Triton X-100 in PBS (PBST), and blocked for at least 2 h with 6% BSA in PBST at room temperature. They were then incubated at 4 °C overnight with the following antibodies: mouse anti-SOX17 (1:2000; OriGene Technologies, USA), rabbit anti-FOXA2 (1:500; Cell Signaling Technology, USA), rabbit anti-OCT4 (1:500; Cell Signaling Technology, USA), guinea pig anti-PDX1 (1:1000; Abcam, Cambridge, UK), mouse anti-NKX6.1 (1:2000, DSHB), goat anti-SOX9 (1:500, R&D Systems), mouse anti-NKX2.2 (1:2000, DSHB), sheep anti-NGN3 (1:1000, R&D Systems), rabbit anti-Chromogranin A (1:4000, Sigma), mouse anti-AFP (1:1000, Sigma), E-cadherin (1:1000, Cell Signaling Technologies), and mouse anti-Ki67-647 conjugate (1:50, BD Biosciences). The cells were washed three times with tris-buffered saline with 0.3% Tween 20 (TBST) and then incubated with the following secondary antibodies: Alexa Fluor 488-labeled anti-guinea pig IgG, Alexa Fluor 568-labeled anti-mouse IgG, Alexa Fluor 488-labeled anti-rabbit IgG, or Alexa Fluor 568-labeled anti-goat IgG (1:500; Molecular Probes, ThermoFisher Scientific). Nuclei were counterstained with Hoechst 33342 (1 μg/ml; ThermoFisher Scientific, USA). The plates were examined by inverted fluorescence microscopy (Olympus) and the images were processed using the Adobe Photoshop software. The antibody details are listed in Table 1.

**Table 1:** Primary antibodies used for immunostaining and FACS

Antibody	Company	Catalog no.	Dilution
Anti-AFP	Sigma	A-8452	1:2000
Anti-Chromogranin A	Sigma	MA5-14536	1:4000
Anti-FOXA2	Cell Signaling	3143	1:200
Anti-Ki67	BD Pharmingen	561126	1:50
Anti-NGN3	R&D Systems	AF3444	1:1000
Anti-NKX6.1	DSHB	F55A12	1:2000
Anti-NKX6.1	R&D Systems	LS-C124275	1:100
Anti-NKX2.2	DSHB	74.5A5	1:2000
Anti-OCT4	Cell Signaling	C30A3	1:500
Anti-PDX1	Abcam	ab47308	1:1000
Anti-PDX1	Abcam	ab7383	1:100
Anti-SOX9	R&D Systems	AF3075	1:500
Anti-SOX17	OriGene	CF500096	1:2000

### Reverse transcription polymerase chain reaction (PCR) and real-time PCR

RNA was extracted using the Qiagen Miniprep RNA extraction kit (Qiagen) and cDNA was synthesized using the superscript™ IV, first strand synthesis system kit (ThermoFisher Scientific, USA). For real-time PCR, we used the SYBR Green-based detection system (GoTaq qPCR Master Mix, Wisconsin, USA) and amplification was detected using Quant Studio 7 system (Applied Biosystems, CA, USA) in triplicate for each protocol. Average Ct values for each protocol were normalized to average Ct for P1-ND and fold-change in gene expression for each protocol was determined with respect to P1-ND. Primer details are listed in Table 2.

**Table 2:** Primer sequences for real-time polymerase chain reaction

Gene	Primer sequence
*PDX1*	5’-CGTCCAGCTGCCTTTCCCAT-3’
	5’-CCGTGAGATGTACTTGTTGAATAGGA-3’
*NKX6.1*	5’-GGGCTCGTTTGGCCTATTCGTT-3’
	5’-CCACTTGGTCCGGCGGTTCT-3’
*SOX9*	5’-GACTACACCGACCACCAGAACTCC-3’
	5’-GTCTGCGGGATGGAAGGGA-3’
*FOXA2*	5’-GGGAGCGGTGAAGATGGA-3’
	5’-TCATGTTGCTCACGGAGGAGTA-3’
*HNF6*	5’-GGACCTCAAGATAGCAGGTTTAT-3’
	5’-CAGAATGCAGGTGAGCTAAGT-3’
*HNF1β*	5’-ACACACCTCCCATCCTCAAG-3’
	5’-CATTTTAGCAGCCCTCCAAG-3’
*NGN3*	5’-GGCTGTGGGTGCTAAGGGTAAG-3’
	5’-CAGGGAGAAGCAGAAGGAACAA-3’
*NEUROD1*	5’-GCCCCAGGGTTATGAGACTAT-3’
	5’-GAGAACTGAGACACTCGTCTGT-3’
*NKX2.2*	5’-GGCCTTCAGTACTCCCTGCA-3’
	5’-GGGACTTGGAGCTTGAGTCCT-3’
*AFP*	5’-ACAGAGGAACAACTTGAGGCTGTC-3’
	5’-AGCAAAGCAGACTTCCTGTTCCTG-3’
*ALB*	5’-GTGAAACACAAGCCCAAGGCAACA-3’
	5’-TCAGCCTTGCAGCACTTCTCTACA-3’

### Flow cytometry

Differentiated cells at specific stages were fixed with 70% ethanol and blocked overnight with 6% BSA in PBST. Cells were stained with the primary antibodies goat anti-PDX1 (1:100; Abcam), goat anti-SOX9 (1:50; R&D Systems), mouse anti-NKX6.1 (1:100; DSHB), goat anti-NKX6.1 (1:50; R&D Systems), and mouse anti-Ki67-647 conjugate (1:50; BD Biosciences) for 3–4 h at room temperature. The cells were incubated with Alexa-fluor secondary antibodies (1:200; Molecular Probes, ThermoFisher Scientific) for 40 min at room temperature. The results were analyzed using the BD Accuri C6 flow analyzer and the results were processed using FlowJo.

### Cell cycle and proliferation assay

To determine the proliferation of differentiated cells generated from the different protocols, the cells were incubated with BrdU (1:100; ThermoFisher Scientific, USA) for 6 h in the differentiation media. DNA synthesis and cell cycle analyses were performed as previously described [29, 30]. Briefly, the cells were dissociated using TrypLE, washed with PBS and then fixed with 70% ethanol overnight. Fixed cells were denatured with 2 M HCl containing 0.5% Triton and neutralized by 0.1 M sodium borate followed by incubation with anti-BrdU antibody (1:100; ThermoFisher Scientific, USA) in 2% BSA in PBS (1:100; ThermoFisher Scientific) for 2 h at room temperature. Cells were then treated with propidium iodide (50 μg/ml) and RNase A solution (250 μg/ml) for 45 mins in the dark at room temperature and finally analyzed using the BD Accuri C6 flow analyzer (BD Biosciences, USA). The results were processed using FlowJo.

## Results

### Optimization of the differentiation protocol to generate PDX1^+^/NKX6.1^+^ pancreatic progenitors

Pancreatic lineage cells originate from the definitive endoderm (DE) layer. In order to maximize pancreatic specification, it is important to obtain a high proportion of DE. We induced DE differentiation following a previously published protocol with some modifications, as described in Fig. 1a. Immunostaining analysis showed high co-localization of the DE-specific TFs SOX17 and FOXA2 (Fig. 1b), with negligible or no expression of pluripotency marker OCT4 (Fig. 1c). In all experiments, we were able to consistently generate above 94% SOX17^+^ endodermal cells that were further directed towards PDX1^+^/NKX6.1^+^ progenitors (Fig. 1d).

**Fig. 2:**
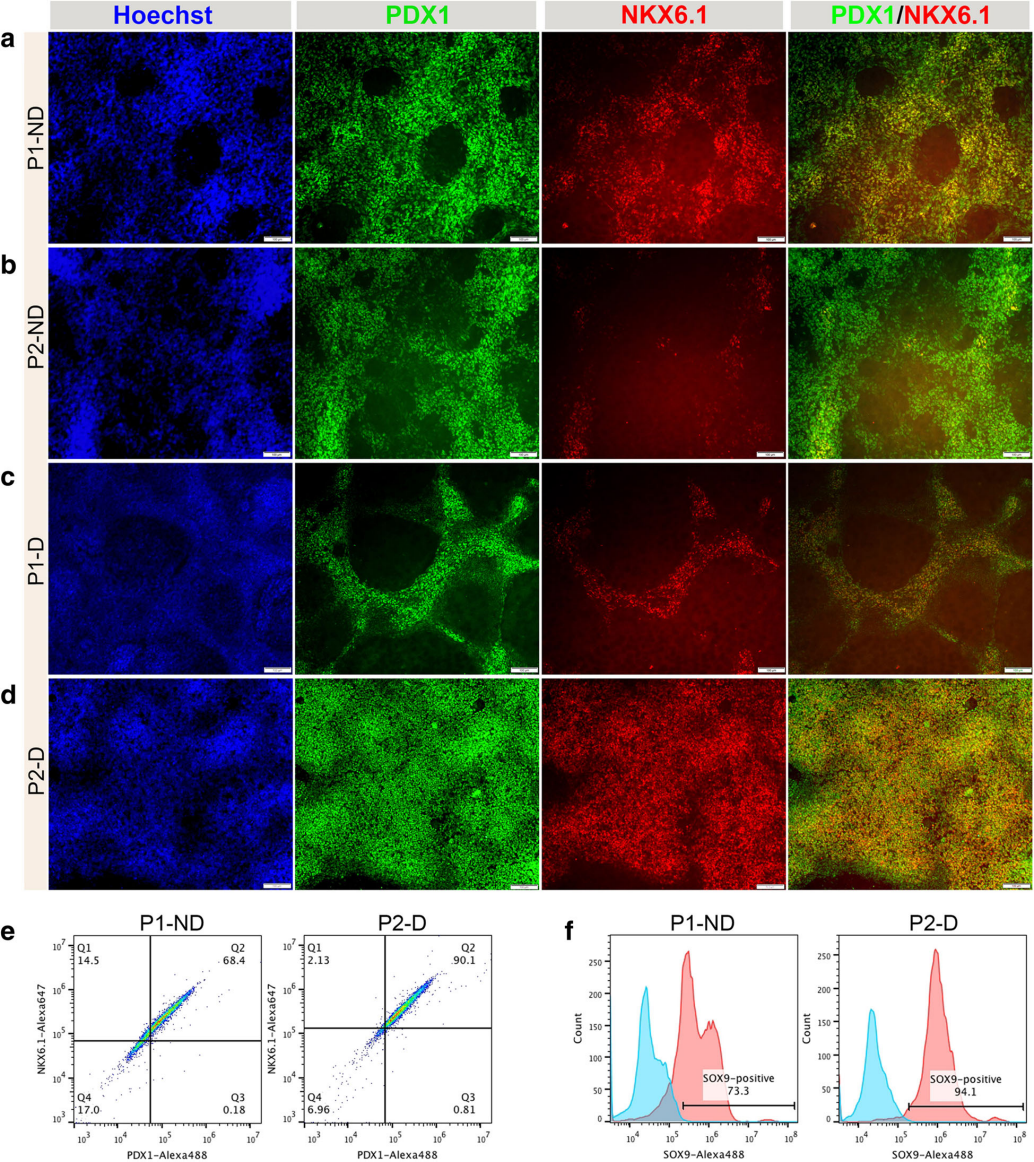
Generation of PDX1^+^/NKX6.1^+^ pancreatic progenitors using different protocols. Immunostaining of PDX1 (green) and NKX6.1 (red) in hESC-derived pancreatic progenitors generated from protocol 1 non-dissociated (P1-ND) (**a**), protocol 2 non-dissociated (P2-ND) (**b**), protocol 1 dissociated (P1-D) (**c**), and protocol 2 dissociated (P2-D) (**d**). Note the high co-expression level of PDX1 and NKX6.1 in the P2-D in comparison to other protocols. Nuclei are labeled with Hoechst. **e** Flow cytometry analysis for the expression of PDX1 and NKX6.1 after stage 4 of differentiation. **f** Flow cytometry analysis for the expression of SOX9 after stage 4 of differentiation. Scale bars = 100 μm

**Fig. 3:**
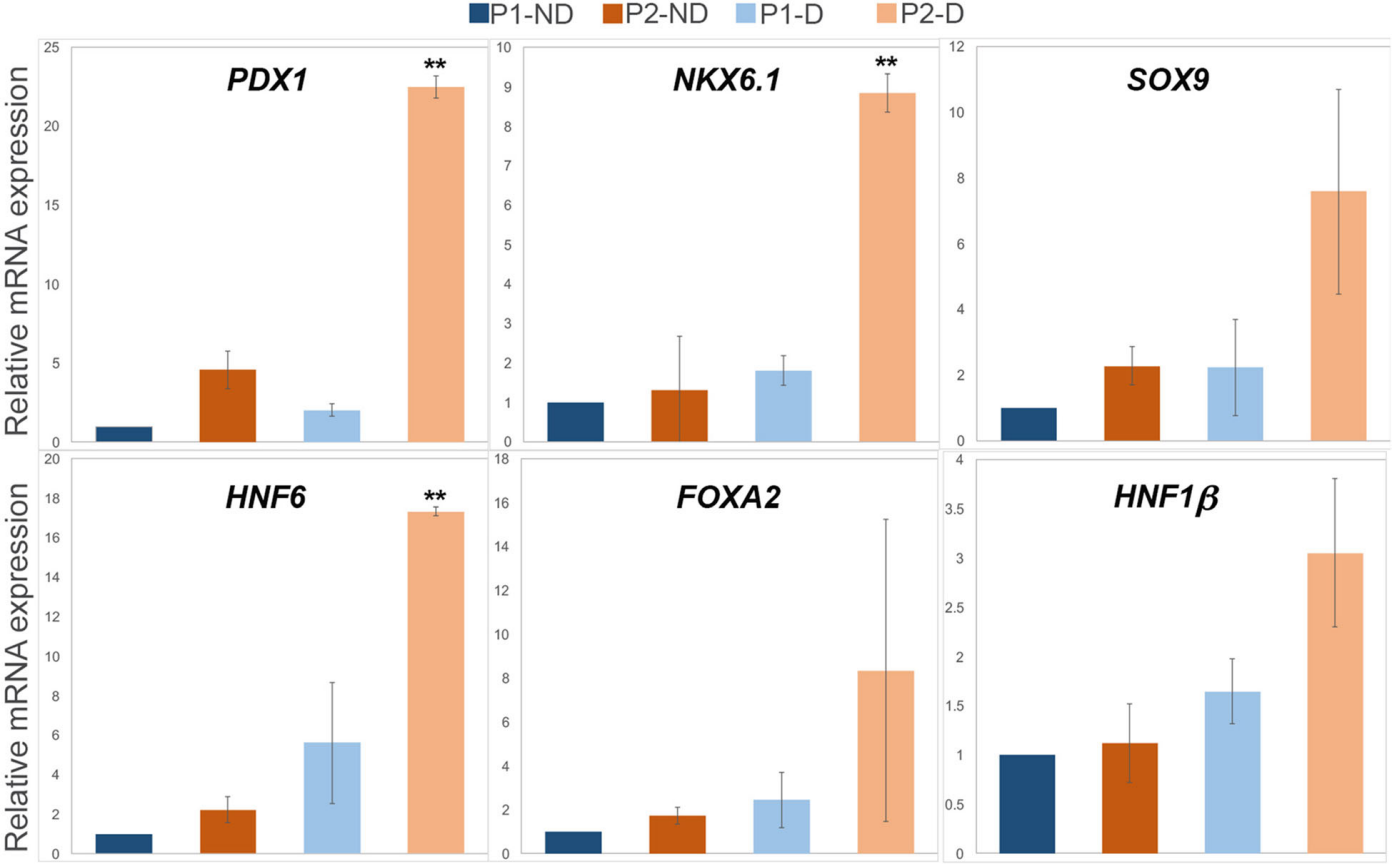
mRNA expression of pancreatic-related transcription factors in pancreatic progenitors generated using different protocols. Real-time PCR analysis for the indicated transcription factors in H1-hESC-derived pancreatic progenitors (at the end of stage 4) generated from protocol 1 non-dissociated (P1-ND), protocol 2 non-dissociated (P2-ND), protocol 1 dissociated (P1-D), and protocol 2 dissociated (P2-D). Relative mRNA expression levels were normalized to baseline expression of these TFs in hESC-derived pancreatic progenitors derived from P1-ND. Relative expression is represented as the mean ± SEM (*n* = 2); **p < 0.01

To generate PDX1^+^/NKX6.1^+^ pancreatic progenitors, first we reproduced the two protocols established by Nostro et al. [19] with some modifications, as indicated in Fig. 1a. Consistent with their results, we demonstrated that changing the length of stage 3 resulted in generation of two types of pancreatic progenitors [19]. Shortening stage 3 to 2 days led to generation of pancreatic progenitors expressing PDX1 and NKX6.1 (PDX1^+^/NKX6.1^+^) (protocol 1-non-dissociated; P1-ND) (Fig. 2a). However, extending stage 3 to 4 days resulted in generation of PDX1^+^/NKX6.1^–^ pancreatic progenitors (protocol 2-non-dissociated; P2-ND) (Fig. 2b). We noticed that although the extension of stage 3 produced a large number of PDX1^+^/NKX6.1^–^ cells, a small number of PDX1^+^/NKX6.1^+^ cells were also seen in culture (Fig. 2b). In order to increase the size of the generated PDX1^+^/NKX6.1^+^ population in P1-ND, we sought to dissociate and re-plate the endodermal cells on day 4 of differentiation (after stage 1) (Fig. 1a). The endodermal cells were dissociated into single cells and were re-plated as monolayer onto new Matrigel-coated plates at half the endoderm density, where the total number of endodermal cells were distributed into at least half their number per plate. These cells were subjected to the same protocols (P1 and P2) that generated the above-mentioned populations (PDX1^+^/NKX6.1^+^ and PDX1^+^/NKX6.1^–^). For each of these protocols, we compared the efficiency of pancreatic fate induction between the non-dissociated (P1-ND and P2-ND) and those dissociated (P1-D and P2-D) (Figs. 2 and 3). Using immunostaining, comparing the efficiency of generating PDX1 and NKX6.1 co-positive progenitors between the above protocols revealed that P2-D generated the highest proportion of PDX1^+^/NKX6.1^+^ progenitors (Fig. 2a–d). Re-plating at a density of 2.5–3.5 × 10^5^ cells/cm^2^ on freshly prepared Matrigel (1:50) maximized the co-localization of PDX1 and NKX6.1 in P2-D generated progenitors (Fig. 2d). These numbers can be obtained by re-distributing one well of a six-well plate into two wells of a six-well plate (half of the endoderm density). These results indicate that the efficiency of NKX6.1 induction in P2-D was significantly higher than all other protocols (Fig. 2a–d), and particularly higher than the previously established protocol by Nostro et al. (P1-ND) (Fig. 2a–d).

Furthermore, analysis of the expression of the epithelial marker E-cadherin showed higher expression in PDX1^+^ cells of P2-D compared to other protocols (Additional file 1: Figure S1), indicating higher induction of the pancreatic epithelium (progenitors) in our optimized protocol. Supported by a previous study showing direct regulation of E-cadherin transcription by PDX1 [31], the high induction of pancreatic epithelium in P2-D is attributed to higher PDX1 expression. Moreover, SOX9 expression was found to be co-expressed with NKX6.1 in all protocols, but its co-expression was the highest in P2-D (Additional file 2: Figure S2). We noticed that NKX6.1 expression was only observed in cells aggregated in high density regions in the non-dissociated protocols in contrast to the dissociated protocols where it was observed throughout the re-plated cells at low density (Additional file 1: Figure S1 and Additional file 2: Figure S2).

Since P1-ND and P2-D generated reasonable numbers of PDX1^+^/NKX6.1^+^ cells (Fig. 2a, d), we quantified the co-expression levels of PDX1 and NKX6.1 using flow cytometry in P1-ND and P2-D. The percentage of cells that co-expressed PDX1 and NKX6.1 was dramatically higher in P2-D (~90%) in comparison to the P1-ND protocol (~68%) (Fig. 2e). Likewise, SOX9 expression was dramatically upregulated in P2-D (~94%) in comparison to P1-ND (~73%) (Fig. 2f). These results combined indicate that our optimized protocol (P2-D) efficiently induces high PDX1 and NKX6.1 co-expressing pancreatic progenitors in monolayer.

Next, we performed real-time PCR to quantify the mRNA levels of pancreatic TFs at the end of stage 4 for the different protocols. The mRNA analysis showed a significant upregulation of PDX1 and NKX6.1 TFs in pancreatic progenitors differentiated using P2-D in comparison to all other protocols (Fig. 3). Furthermore, the expression levels of key pancreatic development TFs, including SOX9, HNF6, FOXA2, and HNF1-β, were dramatically increased in dissociated cells, with a higher fold increase in those differentiated using P2-D than all other protocols (Fig. 3). These results indicate that dissociation after endoderm formation followed by a longer induction with stage 3 cytokines (P2-D) enhances generation of PDX1^+^/NKX6.1^+^ pancreatic progenitors, which are the bona fide source for functional pancreatic β cells [10].

### Culturing of dissociated endodermal cells at lower density generates a novel PDX1^–^/NKX6.1^+^ population

**Fig. 4:**
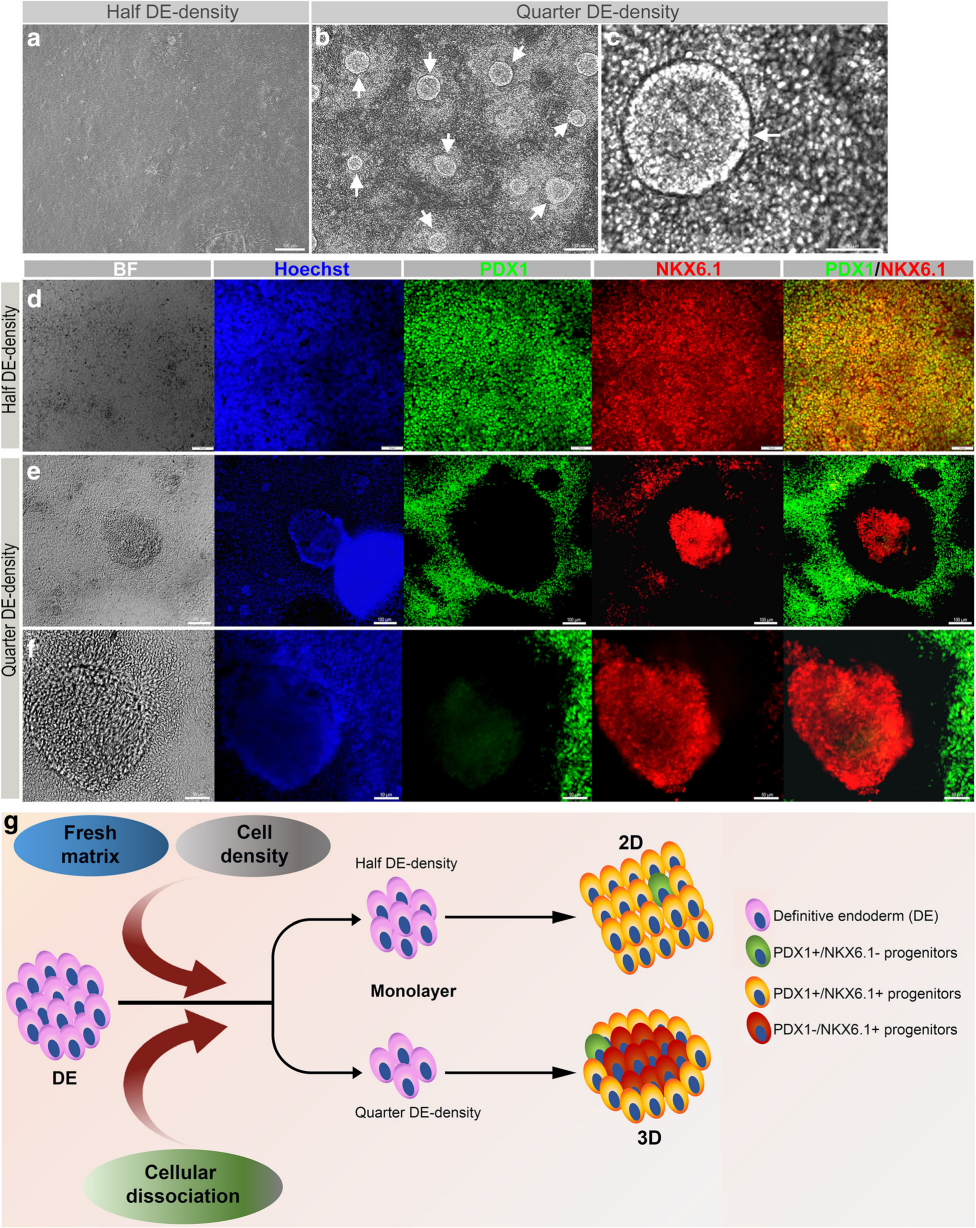
Effect of cell density on PDX1 and NKX6.1 co-expression in protocol 2-dissociated pancreatic progenitors. **a**–**c** Differences in morphology of pancreatic progenitors dissociated and re-plated at either half or quarter of the definitive endoderm (DE) density. Note the 3D structures that appeared at the quarter DE density re-plated culture (**b**, **c**). Generation of high PDX1^+^/NKX6.1^+^ co-positive population in half DE density (**d**) and a novel PDX1^–^/NKX6.1^+^ population in quarter DE density re-plated culture (**e**, **f**). **g** Graphical summary for generation of the monolayer PDX1^+^/NKX6.1^+^ population and compact three-dimensional (3D) structures comprising of a PDX1^–^/NKX6.1^+^ population at a half and a quarter of the endoderm re-plating densities. The generation of the two pancreatic progenitor populations is a result of a combined effect orchestrated by endodermal cell dissociation, cell density, and extracellular matrix on pancreatic differentiation. All data shown are representative results from at least three independent experiments. Scale bars in **a**, **b** and **e** = 50 μm and **d**, **c** and **f** = 50 μm

To further optimize the efficiency of the P2-D protocol to generate PDX1^+^/NKX6.1^+^ cells, we evaluated the effect of re-plating the dissociated cells at different cell densities (Fig. 4). As mentioned above, re-plating at a density of 2.5–3.5 × 10^5^ cells/cm^2^, which was on average about half the density of the generated endodermal cells, gave the highest efficiency for generation of PDX1^+^/NKX6.1^+^ cells using P2-D (Figs. 2d–f, 3, and 4a, d). Interestingly, dissociating and re-plating the cells at about one-quarter of the endodermal density (1.0–1.5 × 10^5^ cells/cm^2^) resulted in the appearance of three-dimensional (3D) structures (Fig. 4b, c). These structures were compact with well demarcated borders and surrounded by monolayer cells (Fig. 4b, c). Immunostaining analysis of the half- and quarter-density re-plated cells showed that most of the monolayer cells were co-positive for PDX1 and NKX6.1 (Fig. 4a, d), while most of the 3D structures that appeared in quarter-density cultures were only positive for NKX6.1 (PDX1^–^/NKX6.1^+^) (Fig. 4e–g). These results were reproduced from several independent experiments using different hPSC lines (H1-hESCs and IMR90-hiPSCs) (Fig. 4g).

We have evaluated the potential of this population to be specified towards the endocrine lineage. Interestingly, we saw that some of these PDX1^–^/NKX6.1^+^ structures co-expressed the endocrine marker NKX2.2, while all of them were negative for the master regulator of endocrine progenitors, NGN3 (manuscript submitted for publication). However, one unexpected observation was the absence of chromogranin A (CHGA) in this population. Our findings strongly suggest that they are a novel pancreatic progenitor population and are currently under characterization (manuscript submitted for publication). These findings indicate that re-plating the endodermal cells at a very low density on fresh Matrigel can generate a novel population expressing NKX6.1 in the absence of PDX1 (PDX1^–^/NKX6.1^+^), which may have the potential to generate functional pancreatic β cells.

### The optimized pancreatic differentiation protocol increases cell proliferation

**Fig. 5:**
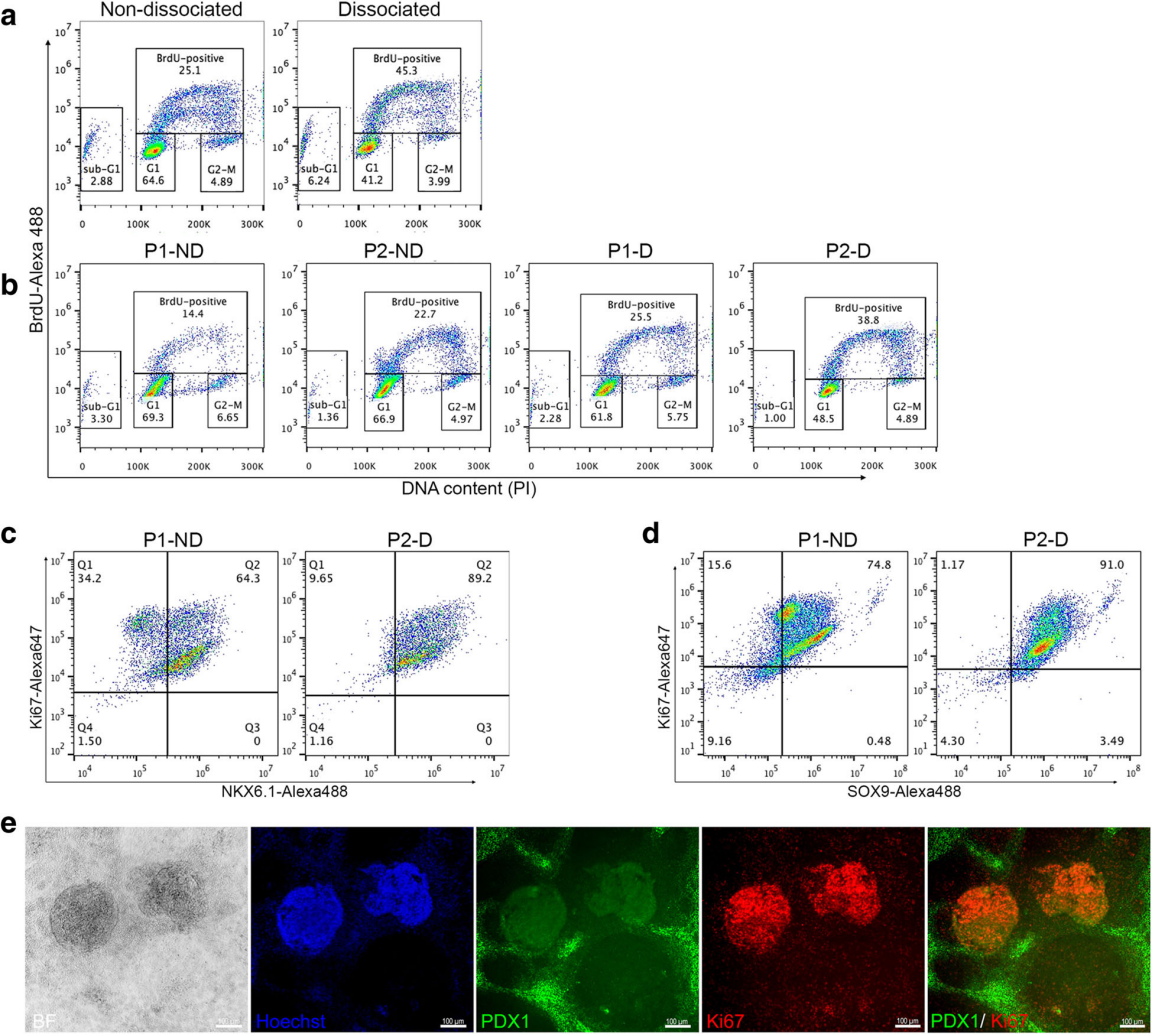
Enhancement of pancreatic progenitor differentiation is associated with an increase in cell proliferation. Flow cytometry analysis of BrdU incorporation (6 h) and DNA content in dissociated and non-dissociated cells at day 5 (**a**) and day 10 (**b**) of differentiation into pancreatic progenitors. Flow cytometry analysis for the co-expression of Ki67 (proliferation marker) with NKX6.1 (**c**) and SOX9 (**d**) after stage 4 of differentiation in P1-ND and P2-D progenitors. Double immunofluorescence staining for Ki67 and PDX1 (**e**) in novel pancreatic progenitors generated from P2-D protocol. Scale bars = 50 μm. P1-D protocol 1 dissociated, P1-ND protocol 1 non-dissociated, P2-D protocol 2 dissociated, P2-ND protocol 2 non-dissociated

Even though the dissociated cells were re-plated in smaller numbers than the non-dissociated cells, we observed that the densities of the cells in P1-D and P2-D were high at the end of stage 4 suggesting an increase in proliferation of the dissociated cells. To investigate whether the improvement in the differentiation efficiency is associated with alterations in the cell proliferation profiles, we analyzed the DNA synthesis and cell cycle at different time points during the differentiation into pancreatic progenitors. To measure the DNA synthesis, the cells were exposed to BrdU incorporation for 6 h. At the end of stage 2 (day 5 of differentiation), flow cytometry analysis for BrdU and DNA content showed an increase in BrdU incorporation in dissociated cells in comparison with those of non-dissociated cells (Fig. 5a). Furthermore, analyzing the cells of the four protocols at day 9 of differentiation showed a marked increase in DNA synthesis in cells of P2-D in comparison with the other protocols (Fig. 5b). Consistent with the BrdU incorporation results, cell cycle analysis showed that the distribution of cells in G1, S, and G2/M phases was altered. A reduction in the proportion of cells in G1 phase and an increase in the proportion of cells in S (DNA synthesis) phase was observed in the dissociated cells at day 5 of differentiation in comparison with non-dissociated cells (Fig. 5a). Likewise, at day 9 of differentiation, there was a dramatic reduction in the proportion of cells in G1 phase and an increase in the proportion of cells in S phase in P2-D progenitors compared to the other protocols (Fig. 5b). These findings indicate that the combination of dissociation and re-plating endodermal cells with extension of the duration of stage 3 (P2-D protocol) increases the DNA synthesis rate and cell cycle progression.

We next gauged the proliferative activity of the PDX1^+^/NKX6.1^+^ population generated from our optimized P2-D protocol using the proliferation marker Ki67. Our results showed that the percentage of cells co-expressing NKX6.1 and Ki67 was higher in P2-D (~89%) in comparison to that of P1-ND (~64%), indicating that our optimized P2-D protocol elevates the proliferative capacity of NKX6.1^+^ cells (Fig. 5c). Furthermore, we consistently recorded above 90% co-localization of SOX9 with Ki67 in the P2-D protocol, which was higher than in P1-ND (~74%) (Fig. 5d). To assess the proliferative capability of the 3D structures generated by re-plating at a quarter of the endoderm density that expressed NKX6.1 (PDX1^–^/NKX6.1^+^ cells), we co-stained them with Ki67 and PDX1. Our results showed that the 3D structures (PDX1^–^/NKX6.1^+^ cells) exhibited highly proliferative activity in comparison with other surrounding PDX1^+^ progenitor cells (Fig. 5e). As expected, these structures were negative for PDX1 expression (Fig. 5e). Taken together, these findings indicate that our optimized protocol generates highly proliferative multipotent pancreatic progenitors (PDX1^+^/NKX6.1^+^ and PDX1^–^/NKX6.1^+^).

### Enhancement of pancreatic progenitor differentiation is associated with inhibition of early hepatic markers

**Fig. 6:**
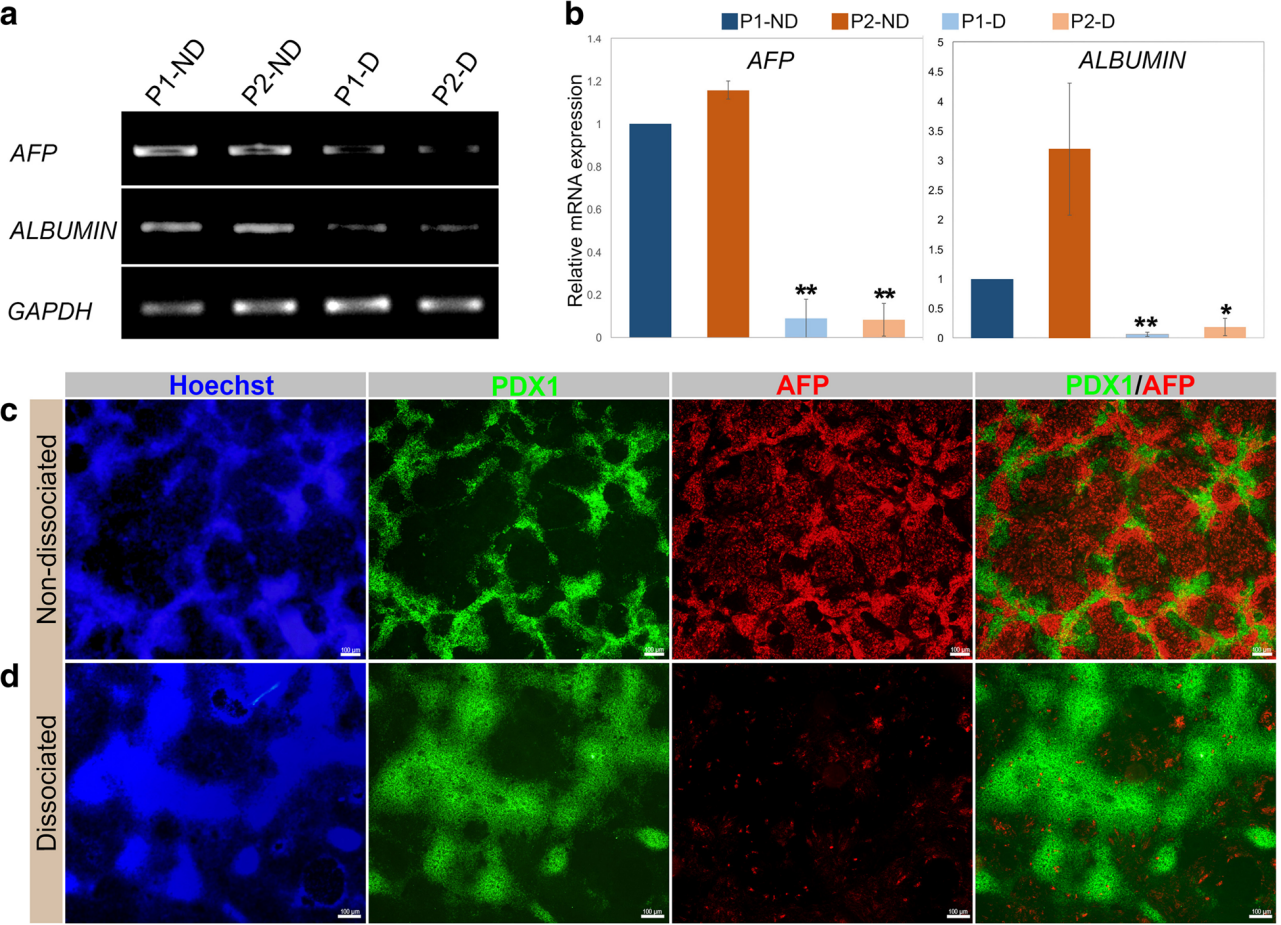
Optimized protocol-derived pancreatic progenitors suppress hepatic fate. **a**, **b** mRNA expression of alpha-fetoprotein (AFP) and ALBUMIN in pancreatic progenitors generated using different protocols at the end of stage 4 of differentiation. Immunofluorescence staining for AFP and PDX1 for **c** non-dissociated protocol 1 (P1-ND) and **d** dissociated protocol 2 progenitors (P2-D). Nuclei are labeled with Hoechst. All data shown are representative results from at least three independent experiments. P1-D protocol 1 dissociated, P2-ND protocol 2 non-dissociated

In order to further characterize the efficiency of our optimized protocol, we investigated the effect of dissociation on hepatic specification since pancreatic and hepatic progenitors commonly bud from the DE layer. mRNA levels of key hepatic markers such as alpha-fetoprotein (AFP) and ALBUMIN were remarkably decreased in dissociated progenitors (P1-D and P2-D) compared with the non-dissociated protocols (P1-ND and P2-ND) (Fig. 6a, b). Furthermore, immunostaining results showed that AFP protein was dramatically downregulated in P2-D in comparison with P1-ND (Fig. 6c, d). This suggests that the dissociation of endoderm dramatically inhibits the alternate hepatic lineage by downregulating the expression of early hepatic genes AFP and ALBUMIN, thereby improving the efficiency of pancreatic differentiation.

### Dissociated PDX1^+^/NKX6.1^+^ progenitors generate endocrine progenitors

**Fig. 7:**
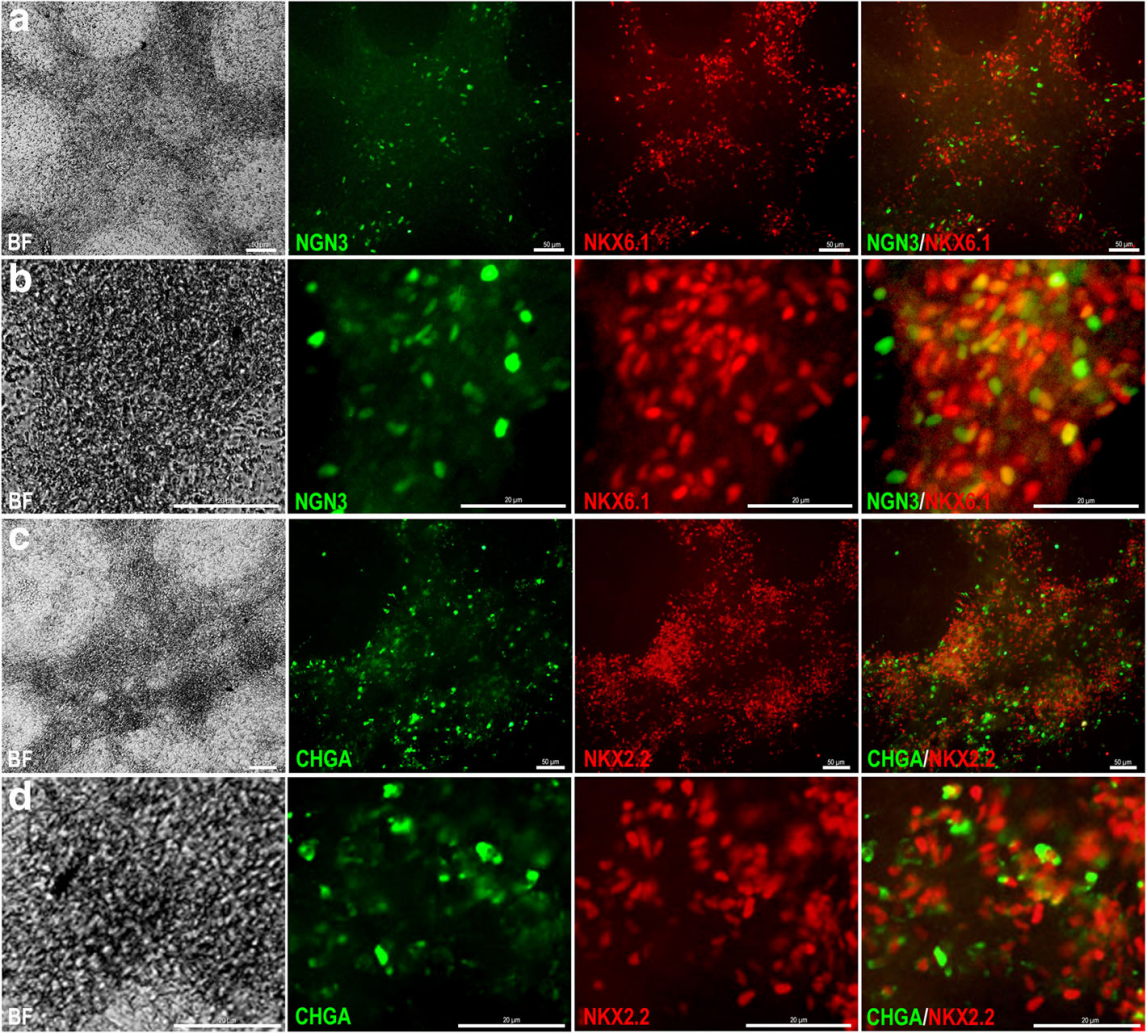
Optimized protocol-derived pancreatic progenitors efficiently generate endocrine progenitors. **a** Immunofluorescence images showing the co-expression of the endocrine marker NGN3 with NKX6.1 at the end of stage 5 (and at higher magnification in **b**). **c** Immunofluorescence images showing the co-expression of endocrine markers CHGA and NKX2.2 at the end of stage 5 (and at higher magnification in **d**). Nuclei are labeled with Hoechst. Scale bars in **a** and **c** = 50 μm and in **b** and **d** = 20 μm

To validate the developmental potential of the PDX1^+^/NKX6.1^+^ cells generated using our protocol (P2-D), we further differentiated the PDX1^+^/NKX6.1^+^ progenitors generated from P2-D into stage 5 to generate endocrine progenitors in adherent monolayer culture (Fig. 7a–d). Immunostaining results showed high expression of endocrine markers such as NGN3, NKX6.1, NKX2.2, and CHGA at the end of stage 5 (Fig. 7a–d). We noticed co-localization of NGN3 with NKX6.1 (Fig. 7a, b) and NKX2.2 with CHGA (Fig. 7c, d), which was not present in all cells. This validates that our P2-D generated PDX1^+^/NKX6.1^+^ pancreatic progenitors that can efficiently generate endocrine precursors in vitro under adherent conditions.

## Discussion

In this study, we provide an efficient protocol for a high induction of PDX1^+^/NKX6.1^+^ pancreatic progenitors in monolayer culture, critical to the generation of functional β cells. We show that dissociation of a densely formed endoderm and re-plating at low density followed by longer retinoid and FGF10 signaling results in a high yield of pancreatic progenitors expressing key markers for pancreatic β-cell development. This enhanced differentiation is complemented by higher proliferation of PDX1^+^/NKX6.1^+^ progenitors during the extended stage 3 treatment as well as being due to impeded alternate hepatic lineage specification.

The significance of cell density and cell-cell contact in promoting pancreatic differentiation has been previously reported, specifically in regulating endocrine differentiation [26]. A previous report showed that cell aggregation and cell-cell contacts stimulate growth of the pancreatic epithelium and increase transcription of pancreatic genes in fetal pancreatic tissue in monolayer culture [25]. In this study, we observed that the PDX1 and NKX6.1 co-expression in non-dissociated protocols is only seen in highly dense regions within the adherent cultures, which is in accordance with the recent findings of Toyoda et al. that PDX1^+^/NKX6.1^+^ is enriched in the cell aggregates and that a high density is necessary for NKX6.1 induction [27]. Of note, the re-plated cells of P2-D proliferated to give a high density at the end of stage 4, indicating that a high cell density may be required at later stages during pancreatic progenitor differentiation. On the other hand, we found that re-plating of the dissociated endodermal cells at a lower density induced NKX6.1 expression in both PDX1^+^/NKX6.1^+^ and PDX1^–^/NKX6.1^+^ populations. Our results for NKX6.1 induction at very low (a quarter that of the endoderm) density within the novel PDX1^–^/NKX6.1^+^ population is in accord with a recent study reporting that NKX6.1^+^ cells can be produced at low-density culture in the presence of ROCK-NM II inhibitor [28].

Interestingly, we found that the novel PDX1^–^/NKX6.1^+^ population re-oriented themselves in well-defined, compact, 3D structures. Formation of 3D cell clusters has been shown to contribute to islet function and maturation [32, 33]. Expression of NKX6.1 independent of PDX1 in these clusters highlights the high likelihood of this population differentiating into endocrine cells since few studies have pointed out the disappearance of PDX1 at certain stages of development of endocrine progenitors [34, 35]. Additionally, expression of NKX2.2, a downstream target of NGN3 [35, 36], strongly suggests that these cells may be specified towards the endocrine lineage following loss of transient NGN3 expression, but are not yet endocrine cells as suggested by the absence of chromogranin A (Aigha et al., manuscript submitted for publication). Moreover, these clusters also showed a high proliferative capacity, as determined by the expression of Ki67. The characteristics of this novel population presented here suggest that these may be multipotent progenitors en route to an endocrine specification. Overall, our results from manipulation of re-plating densities indicate that dissociation of the densely formed endodermal cells facilitates re-arrangement and re-establishment of appropriate cell-cell contact of the re-plated cells, which improves the efficiency of inducing PDX1^+^/NKX6.1^+^ and PDX1^–^/NKX6.1^+^ pancreatic progenitors, both of which are proposed to be β-cell precursors.

Nonetheless, re-plating the dissociated endodermal cells on a fresh Matrigel matrix may also have beneficial effects on further differentiation into pancreatic progenitors. The crucial role played by the ECM in directing stem cells towards a specific fate has been previously reported [37, 38]. Matrigel, in particular, along with its core component laminin, was shown to have a pro-endocrine effect since pancreatic ductal epithelial cells upregulated endocrine markers on an overlay with Matrigel more than with other matrices such as collagen [39]. Therefore, re-plating on fresh Matrigel matrix during differentiation enhanced the induction of NKX6.1 and other endocrine markers in our dissociated protocols. Of note, several studies have reported the influence of ECM components on pancreatic development, regulating diverse aspects of the cell’s physical microenvironment [20–26]. Taken together, our findings indicate the involvement of factors such as the extracellular microenvironment, in addition to the cell density and soluble molecules, in determining pancreatic cell fate during differentiation.

Importantly, our findings showed that an extended stage 3 treatment of 4 days (retinoid, FGF, hedgehog inhibition, and BMP inhibition) induced the highest NKX6.1 expression following dissociation of endoderm, which was higher than those treated for only 2 days. This is in contrast to the recent study that showed that NKX6.1 is predominantly induced by a shorter stage 3 treatment, and its expression is lowest in extended stage 3 (4 days) induced progenitors [19]. It is noteworthy to highlight that dissociation following endoderm trumps the tendency of these differentiated progenitors (P2-D) with extended stage 3 treatment to prevent NKX6.1 expression and differentiate into PDX1^+^/NKX6.1^–^ cells (P2-ND, as per Nostro et al. [19]) and instead increases overall NKX6.1 mRNA and protein levels. Although our cytokine and growth factor cocktail for all stages was highly similar to the protocol of Nostro et al. [19], with the exception of the dissociation and re-plating steps, the results for pancreatic gene expression, particularly NKX6.1, were different. This could shed light on the dissociation-induced alteration in the physical environment of the cells, implicating modulation of the extracellular microenvironment and cell-cell contact as key players in pancreatic differentiation enhancement.

Retinoid and FGF signaling are activators for PDX1 in the pancreas [18]; therefore, the upregulation of PDX1 expression in the generated progenitors is as a result of extended retinoic acid and FGF signaling, which was enhanced by endodermal cell dissociation. Furthermore, the enhancement of PDX1 expression following dissociation of the endodermal cells is supported by the previously demonstrated inductive effect of dissociating endoderm before differentiating into pancreatic progenitors in improving PDX1 expression [40]. However, in addition to dissociation, our results also implicate retinoid and FGF signaling in inducing NKX6.1 expression since stage 3 extension (P2-D) showed higher NKX6.1 expression than P1-D, while the duration of nicotinamide and EGF treatment (stage 4), which are the known inducers of NKX6.1 [19], remained the same across all protocols.

Both pancreatic and hepatic progenitors originate from DE cells during development and it has been reported that PDX1^+^ pancreatic precursors develop at the expense of hepatic progenitors [10, 41]. In vitro pancreatic differentiation protocols employ an inhibitor of hepatic specification (for example, Noggin) to enhance pancreatic fate specification [5, 18, 42]. Our method of dissociating endoderm, in turn, dramatically reduced the expression of the hepatic markers AFP and ALBUMIN compared to the non-dissociated cells, highlighting the enhancement of pancreatic specification by our technique. In agreement with our results, a recent study reported that PDX1 binds and inhibits the expression of hepatic markers in hESC-derived pancreatic progenitors [43]. This finding suggests that in addition to BMP inhibition using soluble molecules, dissociation that alters cell density and cell-cell contact, and the ECM are essential players in inhibiting hepatic differentiation during pancreatic specification.

Expansion of pancreatic progenitors is a necessity for scaling up their production for clinical use. Since pancreatic progenitors are multipotent cells [10, 41] it is important to optimize in vitro protocols to tap into their multipotency to enhance their proliferation. Our data showed that dissociation and re-plating at half the endodermal density increased cell proliferation by promoting G1/S transition of the cell cycle during the early stages of differentiation. While a recent study optimized the self-replicating capacity of PDX1^+^/SOX9^+^ progenitors [44], it is crucial to specifically increase the proliferative capacity of NKX6.1^+^ cells to enhance the efficiency of obtaining mono-hormonal endocrine cells. An increased fraction of dissociated cells entering S phase facilitated the generation of ~90% NKX6.1^+^/Ki67^+^ proliferative progenitors at the end of stage 4 in our optimized protocol. Moreover, few studies have reported the essential role of cell proliferation regulators in enhancing pancreatic differentiation [45, 46]. Therefore, an increased co-expression of PDX1 and NKX6.1 (P2-D) may be due to increased proliferation of the progenitor cells during differentiation. Indeed, our optimized method for generating PDX1^+^/NKX6.1^+^ proliferative pancreatic progenitors could facilitate their scalable production for transplantation therapy.

PDX1^+^/NKX6.1^+^ cells generated by our optimized protocol were able to generate NGN3^+^/NKX6.1^+^ endocrine progenitors. The induction of NGN3 expression is essential for pancreatic progenitors to be directed toward the endocrine fate [34, 41]. Specifically, co-localization of NKX6.1 with NGN3 in the same endocrine precursor cell specifically directs the cell towards a pancreatic β lineage [41, 47]. Our endocrine precursors also expressed high levels of CHGA and NKX2.2, validating their potential to differentiate into β cells.

Although the generation of mono-hormonal β cells in vitro has been reported [7–9], the differentiation efficiency and β-cell functionality requires further improvement. To enhance the differentiation efficiency, protocols attempt to mimic the in vivo microenvironment by determining appropriate combination of soluble factors; however, they do not pay close consideration to the in vitro culture conditions represented by the cell’s physical environment that may potentially contribute to improving the differentiation efficiency. Therefore, here we examined the effect of modulating the factors controlling the cell’s physical microenvironment on pancreatic differentiation. The mechanism underlying our observations is not fully understood; however, our results strongly suggest an orchestrated interaction between soluble molecules and the extracellular microenvironment for enhancing pancreatic differentiation efficiency.

## Conclusions

In conclusion, we demonstrated that manipulating the cell seeding density, cell-cell contact, and cues from the extracellular matrix are essential elements in improving pancreatic differentiation efficiency and proliferation, thereby providing a simplified differentiation method for generating pancreatic progenitors in vitro under adherent culture conditions. Our analyses indicate that pancreatic progenitors produced by our protocol (P2-D) efficiently express key markers necessary for generating functional β cells. Our further work will focus on optimizing endocrine differentiation conditions using P2-D-derived PDX1^+^/NKX6.1^+^ and studying pathways regulating β-cell development and functionality. Indeed, our novel method for maximizing PDX1^+^/NKX6.1^+^ progenitors from hPSCs in monolayer culture could serve as a source of highly proliferative pancreatic progenitors, providing a platform for future scalable production of functional β cells in vitro.

## Additional files

Additional file 1: Figure S1. Expression of E-cadherin in pancreatic progenitors generated from different protocols. Double immunofluorescence staining for PDX1 and E-cadherin in pancreatic progenitors generated using different protocols. Note the highest induction of E-cadherin, an epithelial marker, in PDX1^+^ progenitors derived using P2-D. Scale bars = 100 μm. (JPG 3473 kb)

Additional file 2: Figure S2. Co-expression of NKX6.1 and SOX9 in pancreatic progenitors generated from different protocols. Double immunofluorescence staining for NKX6.1and SOX9 in pancreatic progenitors generated using different protocols. Note the highest induction of NKX6.1^+^/SOX9^+^ in progenitors derived using P2-D. Scale bars = 100 μm. (JPG 2484 kb)

## Abbreviations

AFP: Alpha-fetoprotein
bFGF: Basic fibroblast growth factor
BSA: Bovine serum albumin
CHGA: Chromogranin A
DE: Definitive endoderm
ECM: Extracellular matrix
FGF: Fibroblast growth factor
hESC: human embryonic stem cell
hiPSC: Human induced pluripotent stem cell
hPSC: Human pluripotent stem cell
NGN3: Neurogenin 3
NKX6.1: NK6 homeobox transcription factor-related locus 1
P1-D: Protocol 1-dissociated
P1-ND: Protocol 1-non-dissociated
P2-D: Protocol 2-dissociated
P2-ND: Protocol 2-non-dissociated
PBS: Phosphate-buffered saline
PCR: Polymerase chain reaction
PDX1: Pancreatic and duodenal homeobox 1
PP: Pancreatic progenitor
T1D: Type 1 diabetes
T2D: Type 2 diabetes
TF: Transcription factor
