# Correction to: Enhanced differentiation of human pluripotent stem cells into pancreatic progenitors co-expressing PDX1 and NKX6.1

**DOI:** 10.1186/s13287-018-1002-2

**Published:** 2018-10-07

**Authors:** Bushra Memon, Manale Karam, Sara Al-Khawaga, Essam M. Abdelalim

**Affiliations:** 1 Diabetes Research Center, Qatar Biomedical Research Institute, Hamad Bin Khalifa University, Qatar Foundation, Doha, Qatar; 2 Cancer Research Center, Qatar Biomedical Research Institute, Hamad Bin Khalifa University, Qatar Foundation, Doha, Qatar

## Correction

The original article [1] contains a number of small errors which the authors would like to clarify:

1. In Fig. 1a of the original article, FGF10 concentration should be 50 ng/ml – as correctly stated in the Methods section – instead of 100 μg/ml.
2. Also in Fig. 1a of the original article, Activin A and EGF concentrations should both be 100 ng/ml – as correctly stated in the Methods section – instead of 100 μg/ml.
3. In the Methods section under the sub-heading “**Differentiation of human pluripotent stem cells into pancreatic progenitor**”, the term, ‘50 ng/ml hFGF10’ should be omitted from the following sentence:

“*At the end of stage 3, media were changed to DMEM supplemented with 1% vol/vol B27, 0.25 mM vitamin C, 50 ng/ml hFGF10, 50 ng/ml hNOGGIN, 100 ng/ml hEGF, and 10 mM nicotinamide (Sigma, USA) for 4 days of stage 4 treatment for each protocol (Fig. 1a)*.”

FGF10 was only added during stages 2 & 3 of differentiation as correctly depicted in Fig. 1a.

The corrected version of Fig. 1a can be viewed ahead.


