# Supplementary figures and images for: Enhanced differentiation of human pluripotent stem cells into pancreatic progenitors co-expressing PDX1 and NKX6.1
Source: Stem Cell Res Ther. 2018 Jan 23;9:15. doi: 10.1186/s13287-017-0759-z (PMC5781269; doi:10.1186/s13287-017-0759-z)

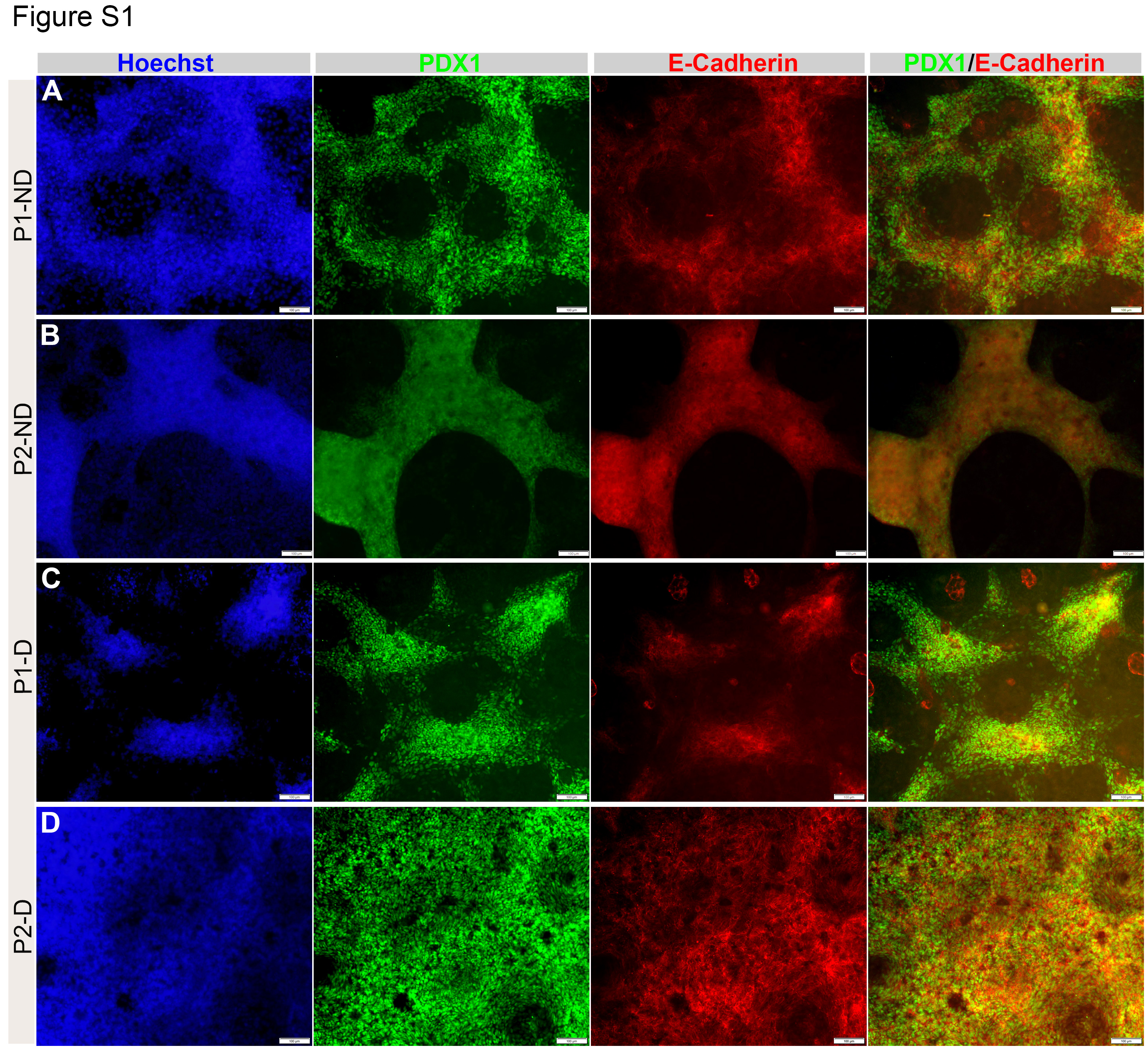

Supplement: Supplementary file 1 — Expression of E-cadherin in pancreatic progenitors generated from different protocols. Double immunofluorescence staining for PDX1 and E-cadherin in pancreatic progenitors generated using different protocols. Note the highest induction of E-cadherin, an epithelial marker, in PDX1+ progenitors derived using P2-D. Scale bars = 100 μm. (JPG 3473 kb) [file 13287_2017_759_MOESM1_ESM.jpg]

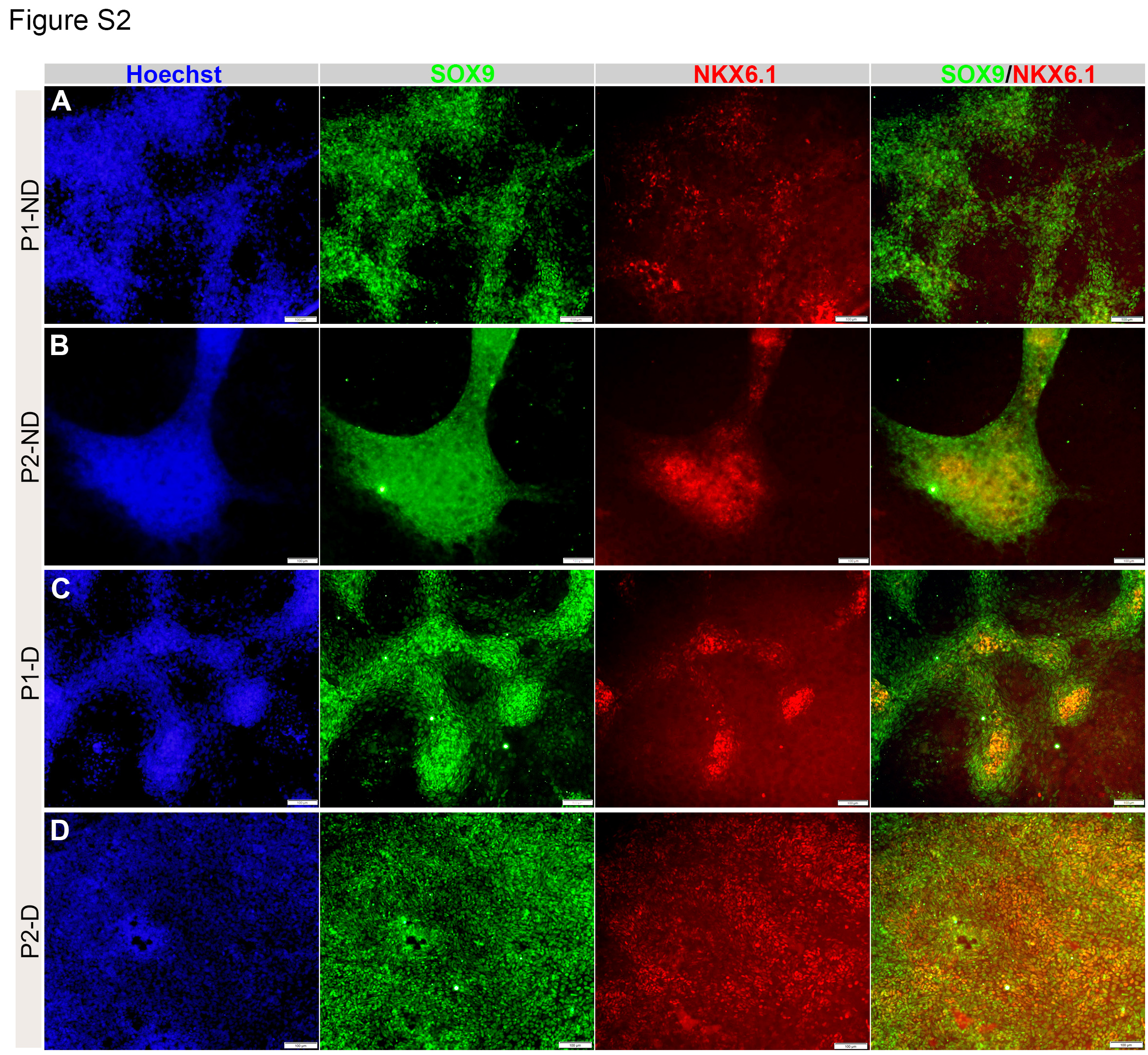

Supplement: Supplementary file 2 — Co-expression of NKX6.1 and SOX9 in pancreatic progenitors generated from different protocols. Double immunofluorescence staining for NKX6.1and SOX9 in pancreatic progenitors generated using different protocols. Note the highest induction of NKX6.1+/SOX9+ in progenitors derived using P2-D. Scale bars = 100 μm. (JPG 2484 kb) [file 13287_2017_759_MOESM2_ESM.jpg]
